# Accessing care services after sexual violence: A systematic review exploring experiences of women in South Africa

**DOI:** 10.4102/curationis.v46i1.2405

**Published:** 2023-10-25

**Authors:** Moreoagae B. Randa, Julie McGarry, Sarah Griffiths, Kathryn Hinsliff-Smith

**Affiliations:** 1 Department of Public Health, Faculty of Health Care Sciences, Sefako Makgatho Health Sciences University, Pretoria, South Africa; 2 Health Sciences School, University of Sheffield and Sheffield Teaching Hospitals NHS Foundation Trust, Sheffield, United Kingdom; 3 Leicester School of Nursing and Midwifery, Faculty of Health and Life Sciences, De Montfort University, Leicester, United Kingdom

**Keywords:** COVID-19 pandemic, healthcare, HIV, referral and support pathways, sexual violence, South Africa, survivors, Thuthuzela Care Centres, qualitative review

## Abstract

**Background:**

Sexual violence against women is a global phenomenon. This is a particular issue in South Africa, where it is estimated with evidence provided that up to half of all women will encounter gender-based and/or sexual violence from a partner during their lifetime. Therefore, evidence suggests that addressing the needs of women in South Africa is a priority.

**Objective:**

This qualitative review aimed to explore the experiences of women seeking care from first contact healthcare facilities in South Africa after sexual violence and during follow-up care.

**Method:**

This systematic review was conducted using the PRISMA checklist for systematic reviews and in line with a published protocol (PROSPERO, CRD42019121580) and searched six relevant databases in 2022. A total of 299 sources were screened, with 5 forming the overall synthesis.

**Results:**

Two synthesised themes of women’s experiences emerged at the time of reporting and during attendance at follow-up healthcare services.

**Conclusion:**

South Africa does have an established legal framework for prosecution and can provide support for survivors of sexual violence through established Thuthuzela Care Centres (TCCs). The review identifies that survivors’ needs are not clearly established when seeking medical attention initially nor identifying support or appropriate pathways.

**Contribution:**

The review has the potential to characterise the support available for women, the type and nature of sexual violence and interventions that may be used by healthcare professionals to support survivors especially during follow-up care.

## Introduction

Gender-based violence (GBV), which includes actual or threatened physical, sexual or psychological harm, coercion or derivation of liberty, is a significant global health and societal issue (World Health Organization [WHO] 2017). It has been estimated that one in three women across the world will experience GBV in their lifetime (WHO 2017). Furthermore, as a result of the pandemic, the United Nations (UN) referred to the rise of domestic violence as a ‘shadow pandemic within the pandemic’ urging countries to address this with urgency (UN 2020) or a ‘twin pandemic’ (Dlamini 2021).

Although included within the wider definition of GBV, sexual violence as a separate entity has been defined as the following:

… any sexual act, attempt to obtain a sexual act, unwanted sexual comments or advances, or acts to traffic, or otherwise directed, against a person’s sexuality using coercion, by any person regardless of their relationship to the victim, in any setting, including but not limited to home and work. (WHO 2002)

Clinical forensic centres, which are located next to the hospitals, treat victims who have been exposed to sexual violence. The centres provide positive treatment for victims as well as clinical forensic medical services. The nurses assess, screen and interview the victims and then proceed to conduct the examination. The forensic medical services provided include a medical examination and the collection of quality specimens for laboratory analysis, which leads to an increase in successful prosecutions of perpetrators and provides communities with confidence (Randa & Mokoena 2019).

The impact of sexual violence on the psychological well-being of those who experience harm can be wide-ranging, including behavioural changes, sleep and eating disorders, post-traumatic stress disorder (PTSD), depression and increased risk of attempting or committing suicide (Jina & Thomas 2013; Naidoo 2013; Sepeng & Makhado 2018). In addition, the physical impact may also extend to significant harms such as unwanted pregnancy and sexually transmitted diseases, including hepatitis B and HIV (Jina & Thomas 2013).

Although sexual violence is a global issue, it has been identified as a large-scale problem in South Africa (Moffett 2006). Kaswa (2021) noted that the coronavirus disease 2019 (COVID-19) pandemic saw a major reduction in accessible healthcare facilities by survivors, including TCCs, during the lockdowns. In 2013–2014, 62 649 sexual offence cases (of which 46 253 cases were rape) were reported to the South African Police Service (Abrahams & Gevers 2017). It has also been estimated that up to half of all women in South Africa experience a lifetime history of physical or sexual violence from a partner (Watt et al. 2017).

A localised study in one South African Province (North-West, Rustenburg) reported that one in four women aged between 18 and 49 years had experienced rape in their lifetime. One in five of these women contracted HIV (Lamola 2017). It is more recently reported that during the pandemic in South Africa, one woman was killed every 3 h (Kaswa 2021). Despite all these stated figures, it has also been observed that this is likely to be an underrepresentation of the real numbers of sexual violence against woman and may only be the ‘tip of the iceberg’, as many cases are not formally reported and/or do not enter the criminal justice system (Sebaeng, Davhana-Maselesele & Manyedi 2016; Steinbrenner et al. 2017). Indeed, a nationwide study in South Africa found that only one in nine women reported being raped to the police (Jewkes & Abraham 2002), and many instances of rape or sexual violence are not reported for fear of retrauma and a lack of confidence in the legal system (Jewkes & Abraham 2002; Rohrs 2011), as emphasised in the records review conducted by Kaswa (2021) that reports a dramatic reduction in attendance and reporting by women at one major TCC.

An enormous amount of work has been undertaken to highlight the experiences of survivors of sexual violence, predominately rape (Abrahams & Gevers 2017; Steinbrenner et al. 2017); there is limited research focusing on the healthcare experiences from the viewpoint of women themselves when they first encounter healthcare provision whether that be 1 of the 54 TCCs located across South Africa (Kaswa 2021). Healthcare facilities and healthcare professionals are often the first port of call for many survivors of sexual violence (McGarry & Hinsliff-Smith 2020). They are often custodians for the care and welfare of survivors, as they help to navigate the next steps to support and referral as well as providing necessary medical care.

Sexual violence can have long-term, debilitating effects on the mental health of victims. Delara (2016) shared that one common form of mood disorders that has been observed in female victims is depression. According to Meekers, Pallin and Hutchinson (2013), feelings of fear, depression, dysthymia and panic were reported as mental consequences by women who experienced violence. The sentiments are echoed by Oshodi et al. (2020), who in their study indicated that symptoms of anxiety, depression and PTSD were found to be persistent among adolescents (study participants aged 14–18), underlining the need for long-term support, screening and evidence-based follow-up care.

The provision of support from healthcare facilities can help to alleviate some of the long-term effects of sexual violence (Abrahams & Gevers 2017), with a need for competent and compassionate healthcare professionals (Rohrs 2011). The WHO found that most healthcare workers in primary healthcare settings do not receive adequate mental healthcare training (Kigozi & Ssebunnya 2009). Again, nurses’ lack of knowledge in managing patients with mental health disorders has resulted in the underdiagnosis of most mental health conditions in primary healthcare (Siddiqi & Siddiqi 2007). The study further found that primary healthcare nurses managing patients at the clinic were not adequately qualified to provide all of the services that are provided in clinics that use a one-stop shop approach (Hlongwa & Sibiya 2019).

To explore these issues further, this review focused on the first point of contact and women’s interactions with healthcare services who have been sexually assaulted, in order to identify how care can be improved for survivors of sexual violence in South Africa at this crucial time. Although literature relating to the assessment of South African follow-up services (Abrahams & Gevers 2017; Lamola 2017) and the procedures and protocols for HIV follow-ups is available (Watt et al. 2017), this routine provision is not the focus for this review because failing to meet the needs of women at the initial stage of seeking healthcare assistance is often paramount to their overall recovery. We also acknowledge that there is ongoing work to understand and resolve the poor HIV adherence and attendance at follow-up appointments by authors in the field (e.g., Holton, Joyner & Mash 2018; Rohrs 2011).

In their study, Sepeng and Makhado (2018) indicated that PTSDs and depression are common among survivors seeking healthcare in TCCs post-rape experiences in South Africa. However, one study reported that adult rape survivors who reported rape in TCCs were not given follow-up care to screen and manage PTSD post-rape experiences (Sepeng & Makhado 2019). It is worth noting that mental healthcare management for rape survivors diagnosed with PTSD and depression requires about 12 sessions with the therapist when using treatment modalities such as cognitive behavioural therapy (CBT), exposure therapy (ET), cognitive processing therapy (CPT), et cetera and this type of care is mostly given in specialised care services such as hospitals. Hence, it is impossible to manage rape survivors diagnosed with these disorders in TCCs.

### Objectives

This review aimed to answer the following overarching questions:

- What are the experiences of women in South Africa when initially accessing healthcare facilities and during follow-up care after a sexual violence?
- What are the reported support mechanisms and referral pathways for survivors of sexual violence when attending a healthcare facility in South Africa?

## Method

The methodology for this review complies with the PRISMA guidelines for systematic reviews (Liberati et al. 2009). The review was conducted in accordance with the protocol, which is registered with the International Prospective Register of Systematic Reviews (PROSPERO, CRD42019121580).

### Eligibility criteria

#### Study requirements

For inclusion, studies were required to be based on empirical data, written in English and published in a peer-reviewed journal. To enable a thorough exploration of first-hand experiences, qualitative studies or mixed-method studies with a clear qualitative component with extractable data were included because the review was exploring experiences of survivors. Randomised control trials (RCTs) and non-RCTs and other intervention studies were excluded, as well as reviews and other non-experimental designs. Studies were required to have been carried out in South Africa with no restrictions on publication date.

#### Participants

Participants included in any studies were required to be female over the age of 18 years who had experienced some form of sexual violence, including rape. While subsection 38 of the *South African Children’s Act* states that children aged 12 years and over can consent independently to medical treatment and other key health interventions if they demonstrate ‘sufficient maturity’ (Republic of South Africa 2005), published articles that explicitly focused on children and girls under the age of 18 years were excluded, as they are likely to have different needs. South African law also dictates that under 18-year-olds cannot consent independently to participate in research and require parental consent (Strode et al. 2010). Confidentiality issues may limit the scope of research with younger participants if parents or legal guardians are required to be involved in the consent process (Tsey 2017). Studies that included male survivors were excluded as we were focused on experiences of women survivors. Any studies exploring the impact of domestic violence or other forms of violence (e.g. emotional, financial and psychological assault) were excluded. Articles focusing only on HIV or adherence to post-exposure prophylaxis (PEP) administered as a result of rape were also excluded as the literature in relation to HIV relates to a programme of services regardless of reason for contracting HIV, and for this review, we are interested in locating the evidence on point of initial contact with any services regardless of HIV infection. In addition, within South Africa, HIV and PEP should be referred to as routine regardless of attending a specialist TCC.

#### Information sources

The following six databases were searched: CINAHL, Cochrane Library, Medline, PsycINFO, PubMed and Web of Science. The initial search was conducted in February 2020 and was repeated in March 2021 and again in July 2022, in order to capture the most up-to-date published evidence and included anything related to women’s experiences during the COVID-19 pandemic in South Africa.

For the updated searches, parameters were added to include research published during 2020–2022 only. Database-specific subject headings and keywords relating to the terms ‘sexual violence’, ‘healthcare facility’ and ‘South Africa’ were searched. Boolean logic was applied using AND, OR in order to produce a comprehensive list of relevant research. Reference lists of screened studies meeting the inclusion criteria and relevant published reviews were searched by hand.

### Search strategy

An example of the full Medline search can be seen in Online Appendix 1. This was adjusted for other databases to include database-specific headings, where available, alongside keyword searches of titles and abstracts.

### Data collection, selection and extraction process

Data were managed using RefWorks software. Initial abstract and title searches and the screening for inclusion were conducted using a checklist from the PROSPERO protocol. The second stage of screening of full texts not excluded from the first stage was initially carried out by two reviewers (SG, MBR, JM or KHS). All studies deemed eligible for inclusion or where uncertainty remained over inclusion were reviewed by a third reviewer. Any discrepancies were resolved through discussion until a consensus was reached on all included studies. For the third phase, all reviewers independently extracted the following data using a data extraction sheet: authors; date; title; institute/region of study; publication journal; methodology; setting; number, age and type of participants; type of violence; research aim; themes and summary of findings.

### Quality assessment

Quality assessment for included studies was carried out independently by two reviewers (authors S.G. and K.H.S.) using CASP qualitative checklist from the Critical Appraisal Skills Programme from 2017, with a focus on research design, sampling strategy, data collection, data analysis, findings, reflexivity, ethical issues and research value. No scoring system is used for CASP, and no study was excluded on quality criteria, but this checklist allowed the reviewers to consider reporting, design, ethics and conduct of included studies.

## Results

### Study selection

**FIGURE 1:**
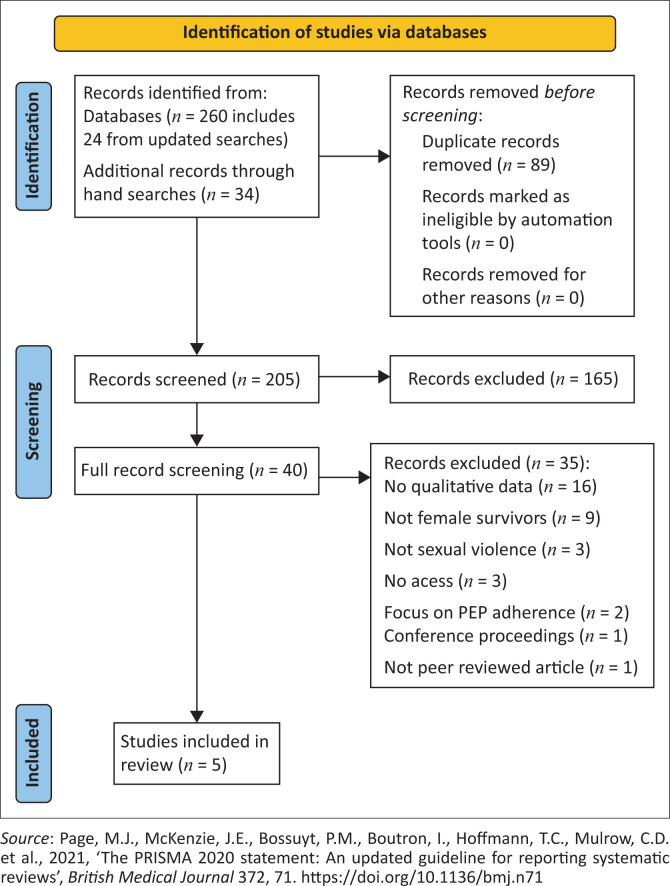
PRISM flow diagram for systematic reviews.

A summary of results from the screening process can be seen in Figure 1. For the first stage of screening, 299 records were screened by abstract and title; after duplicates 210 were screened. Forty-one records were sourced for full-text screening, from which 36 were excluded. Five articles met all inclusion criteria and were included in the review, as shown in Table 1.

**TABLE 1:** Characteristics of included studies.

Author(s), year Location (setting)	Design and method	Participants	Aim of the study	Study’s findings
Abrahams and Gevers (2017) Western Cape (rural and urban)	Qualitative: semi-structured interviews	Rape victims (*n* = 14); service and healthcare providers (*n* = 43) Age: data not presented	To gain a better understanding of acute and long-term (secondary) mental health service provision for rape survivors	Effective and compassionate mental health services should be essential components of post-rape care. Support for providers needs to be strengthened, along with links to ongoing mental healthcare.
Holton et al. (2018) Eden District (hospital settings)	Mixed methods: telephone call surveys with healthcare facilitators; interviews with police and healthcare professionals; semi-structured interviews with rape survivors	Rape victims: girls and women (*n* = 10) Age: 9–42 years (data from 3 participants <18 years not included in this review)	To explore sexual assault survivors’ personal experiences in order to understand barriers and facilitators to attendance at follow-up consultations	Holistic, integrated and patient-centred care is needed to encourage attendance at follow-up appointments. Follow-up care needs to be integrated into chronic care services.
Kaswa (2021). The impact of the COVID-19 pandemic on healthcare service access for the victims of sexual assault	Records review of one TCC in Mthatha regional hospital	Data from 01 January 2014 to 12 December 2020	To review the records to include the period of the South African COVID lockdown in 2020	There was a marked reduction in the number of records indicating that victims did not seek help at the TCC in this study. This saw a drop from approximately 5747 in the period 2014–2020 to only 451 in the 2020 year of the pandemic.
Sebaeng et al. (2016) Rural area, TCC in Mafeking (hospital)	Qualitative: in-depth interviews	Sexual assault: women (*n* = 18) Age: 18–55 years	To explore the experiences of survivors of sexual assault receiving care at a TCC in a hospital within the North-West province	Both formal and informal support are needed towards recovery following sexual assault.
Steinbrenner (2017) Free State Province (Community and university medical care centre)	Qualitative: semi-structured, face-to-face interviews	Sexual assault victims: women (*n* = 6) Age: 22–41 years (mean = 29.3)	To explore the help-seeking experiences of women in South Africa following sexual assault	Women can be reluctant to seek help, and when they do, they may not be provided with what they need to help them recover. There is a need for an integrated service, linking police services, medical care (including prophylactic care) and counselling.

Note: Please see the full reference list of the article, Randa, M.B., McGarry, J., Griffiths, S. & Hinsliff-Smith, K., 2023, ‘Accessing care services after sexual violence: A systematic review exploring experiences of women in South Africa’, *Curationis* 46(1), a2405. https://doi.org/10.4102/curationis.v46i1.2405, for more information.

TCC, Thuthuzela Care Centre.

### Study characteristics

Table 1 provides the study characteristics for included studies. Across the five studies, a total of 48 female sexual violence survivors participated in one study (Abrahams & Gevers 2017). Included in this review was a published article that captured the comparisons of interactions by survivors and a TCC as a major healthcare provider for sexual violence victims pre- and post-COVID-19 pandemic and lockdown in South Africa (Kaswa 2021). This was deemed to be an important work as it provided a useful overview of the situation during the pandemic, and includes services that operated under extensive and protracted lockdown of services. Furthermore, the lack of accessibility to services during that time had a significant effect on reporting and seeking healthcare assistance by survivors.

Four of the five studies used either a purely qualitative paradigm with semi-structured interviews only (Abrahams & Gevers 2017; Sebaeng et al. 2016; Steinbrenner et al. 2017) or mixed methods (Holton et al. 2018). Data from female sexual violence survivors were collected by client intake forms, short questionnaires or telephonic interviews. The studies were conducted in different contexts and locations within South Africa (see Table 1). All studies gathered data directly from survivors of sexual violence, predominately rape, with two studies specifically focusing on women’s lived help-seeking experiences within the South African healthcare system (Sebaeng et al. 2016; Steinbrenner et al. 2017) which provides a useful comparison to the work by Kaswa (2021). The work by Abrahams and Gevers (2017) links to follow-up mental health support in specialist centres, focusing on initial contact with healthcare provision. Where participants under the age of 18 years have accounts attributed to them, we have excluded this from our analysis.

### Thuthuzela Care Centres

In order to understand the findings from included sources, it is important to understand the context in which provision is given for victims of sexual violence in South Africa. The Department of Health in South Africa has 265 healthcare facilities that are designated as providing medical and psychological care and support for victims of sexual violence. Of these, 54 are classed as TCCs that are based in acute settings and are classed as a ‘one-stop shop’, providing legal advice, social assistance, medical care and mental health assessments (Kaswa 2021; Lamola 2017). Thuthuzela is an isiXhosa word meaning ‘comfort’. The purpose of these centres is to provide all health and social care needs for the survivors of sexual violence. They are intended as a victim-centred approach to care, working to a multidisciplinary service delivery model that provides medico-legal services to victims of rape (Bougard & Booyens 2015). Online Appendix 2 provides further details of how TCCs work and what they aim to offer to those who have experienced sexual violence. Within the five sources included in this review, four were conducted in TCCs (Abrahams & Gevers 2017; Holton et al. 2018; Kaswa 2021; Sebaeng et al. 2016) and one with community-based providers (Steinbrenner et al. 2017).

### Quality appraisal

The CASP analysis revealed a lack of explicit consideration for the relationship between the researcher and participants, with only one of the four studies clearly acknowledging this (Steinbrenner et al. 2017). Similarly, only two studies clearly demonstrated consideration of all ethical issues and included ethical approvals (Kaswa 2021; Sebaeng et al. 2016), but three did present rigorous data analysis (Kaswa 2021; Sebaeng et al. 2016; Steinbrenner et al. 2017). One study only provided a very brief overview of findings (Sebaeng et al. 2016). All included studies provided clear aims, appropriately chosen methodologies and appropriate research designs (Abrahams & Gevers 2017; Holton et al. 2018; Kaswa 2021; Sebaeng et al. 2016; Steinbrenner et al. 2017).

### Thematic findings

Table 2 provides the reported themes from the five included studies. Two key themes emerged across included studies: women’s experiences at the time of reporting and women’s experiences during attendance at follow-up healthcare services.

**TABLE 2:** Summary of themes from included studies.

Authors	Main themes	Subthemes
Abrahams and Gevers (2017)	Surviving emotional struggles	-
	Coping	-
	Adherence to HIV prevention medication	-
	Support and care received at rape services	-
	Locating mental health support in acute, post-rape care	-
Holton, Joyner and Mash (2018)	Healthcare system issues	-
	Healthcare provider issues	-
	Police service issues	-
	Community responses	-
	Client factors	-
	Judicial system issues	-
Kaswa (2021)	Reduction in records opened for victims of sexual violence Multiple concerns raised about essential services opening during a pandemic	-
Sebaeng, Davhana-Masalesele and Manyedi (2016)	Lived experiences of sexual assault	Physical trauma
		Psychological trauma
		Interpersonal relationships with opposite gender
	Experiences relating to services received and need for safety and support	Views related to support received
		Views related to safety and insecurity
Steinbrenner, Shawler, Ferreira and Draucker (2017)	Help-seeking in the criminal justice system: fraught justice-seeking	Reluctant reporting to the police
		Fruitless pursuits of justice
	Help-seeking from medical facilities: pragmatic help-seeking	Obtaining needed medical care
		Waiting for service
		Being treated badly/being treated kindly
	Help-seeking from counselling or social services agencies: desperate help-seeking	Seeking relief from emotional anguish
	-	Being cared for and understood
	-	Being able to talk
	-	Moving forward

#### Experiences at the time of reporting

Of the five studies, two focused on the survivors’ experiences when initially reporting a sexual violence crime to authorities (Sebaeng et al. 2016; Steinbrenner et al. 2017). In exploring the South African system and approaches used for the reporting and receiving of any medical attention, there is a dual role to the support offered to survivors from police and healthcare providers. To access the full range of support services, including pregnancy advice, sexual transmitted infections (STIs) information and PEP, survivors are required to report to a TCC within 72 h of the event. It is worth observing that this time frame directly relates to the issue of PEP, which is only effective within 72 h of a possible exposure to HIV (Rohrs 2011). Therefore, it is imperative that survivors either report directly to the police in order to gain the documentation for PEP to be issued or report first to a TCC or hospital for transfer to law enforcement.

Steinbrenner et al. (2017) describe the totality of seeking help from three areas, namely the criminal justice system, healthcare facilities and/or social service agencies. They discussed how the six women survivors in their study described their experiences in each of these contexts, concluding that these can be categorised as ‘fraught justice-seeking’, ‘pragmatic help-seeking’ and ‘desperate help-seeking’. The present system, even with the establishment of TCCs, which have specialist trained staff, is described as wholly inadequate for survivors and can often ‘impede justice and healing rather than facilitate resolutions and recovery’ (Steinbrenner et al. 2017:436).

The work by Sebaeng et al. (2016) focused on one area in the North-West Province of South Africa and one TCC was based in an acute setting. Interviewing, using phenomenological principles, one central question for all the 18 survivors was used:

What is your experience regarding the sexual assault incident that happened to you? (Sebaeng et al. 2016:2)

They categorised women’s accounts of trauma into three areas, namely physical, emotional or psychological and social trauma. The need for safety and support while at the TCC as well as from family and police authorities was highlighted. The survivors were grateful for the support received from TCC staff and felt that this provided the safety and comfort required at this time. However, any good work performed by staff in TCCs can quickly be undermined if the follow-up and dealings with police or responses from family and the wider community are unresponsive, negative or slow to act (Sebaeng et al. 2016).

#### Experiences at follow-up healthcare services

Of the five studies identified in this review, two of the studies (Abrahams & Gevers 2017; Holton et al. 2018) aimed to explore the experiences of women who attended follow-up healthcare services. Abrahams and Gevers (2017) conducted interviews with 14 rape survivors, and they also included 43 service providers. The overarching focus for the aim of the study was to understand the mental health needs of the survivors. The effects of rape and sexual violence are widely understood and acknowledged to have an impact on the survivor’s mental health both in the short and long term (Dworkin & Weaver 2021). Therefore, recognising that survivors will require follow-up interventions from healthcare services is welcomed. These mental health services may include components for a wide range of support, which are linked to stigma, secondary trauma, PTSD or issues relating to pursuing criminal charges, which are well-known factors to influence and affect ongoing recovery for survivors (Chivers-Wilson 2006).

Several studies reported poor integration of mental healthcare services for rape survivors seeking treatment in TCCs in South Africa (Abrahams & Gevers 2017; Petersen et al. 2012). Prosecution of violence against women (VAW) cases in South Africa relies profoundly on oral evidence; hence, testimony is the main form of evidence. A J88 form which is completed by a physician or forensic nurse (Artz & Pithey 2010; Hendry 2010), who have been trained on how to manage VAW clinically but not on how to use legal language, serves as major evidence. The J88 form is the official document from the Department of Justice (Rowe et al. 2013), used exclusively for criminal proceedings, for the purpose of recording injuries sustained by victims of crime, including VAW.

Rees (2010) confirmed that, firstly these results in the J88 forms are being regarded as inaccurate when viewed by the court personnel as the forms neither confirm nor deny the complainant’s allegation. Secondly, the same reports also limit the significance of the J88 in the prosecution of cases, as the health professionals prefer not to draw conclusions of what happened. However, the forensic role of both nurses and emergency care providers in South Africa is still controversial as it is undermined (Mogale, Kushner & Richter 2015). In their study, Sepeng and Makhado (2019) reported that adult rape survivors who reported rape in TCCs were not given follow-up care to screen and manage PTSD post-rape experiences. Engel et al. (2016) indicated that the mental care needed for the survivors is mostly given in specialised care services such as hospitals and are therefore not met in TCCs.

Abrahams and Gevers (2017) strongly indicated that post-rape services, which include mental health provision, are inadequate and scant. Issues cited by survivors in this study included a lack of integrated care provision, spilling over to the nature of the care provided and the feeling of frustration about care provision. One survivor described post-rape care services as ‘a very unrehearsed play’ (Abrahams & Gevers 2017:4). Although survivors in this study expressed gratitude for the post-rape services offered, more work is required to provide the level and type of services that are deemed to meet national or international expectations for the care of survivors. This is particularly pertinent if South Africa is to address the issues of detrimental and long-term mental health issues for the thousands of women and girls who experience sexual violence annually.

Aligned with this is the work by Holton et al. (2018), which described the follow-up care received with a focus on patient-centred care at three hospitals located in the Eden District. All participants reported a positive first contact with healthcare services. However, they cite instances of ‘gatekeeper’ control at follow-up clinics from reception staff or when asked to produce documentation for a follow-up appointment and issues of referral for HIV-positive survivors. Interestingly, it was observed that some survivors were passive to the whole experience and were very much led by healthcare professionals. This was not helped by only providing verbal instructions as opposed to written instructions for follow-up care and support.

## Discussion

The five studies included in this review provide a broad but limited and very sparse view of the experiences of women survivors of sexual violence in South Africa. This review synthesises the limited work that has been conducted within the context of healthcare settings, identifies shortcomings in service provision from the perspectives of survivors and includes empirical studies, which were conducted prior to the global pandemic. We would draw readers to consider work that has been conducted on access to healthcare services by survivors of sexual violence, including work in South Africa during the pandemic, for example, work by Kaswa (2021), which reports the dramatic decrease in reported patient cases in one region of South Africa.

Although only five studies were located, the quality of the reporting, including ethical procedures, provides a useful indicator for the current understanding of sexual violence survivors and their experiences of healthcare provision in South Africa. It is clear that there is much work to be conducted to fully understand the needs of adult female survivors of sexual violence in South Africa. A major problem in understanding women’s experiences is that only 1 in 25 women who experience sexual assault actually report it to the police authorities (Holton et al. 2018) in South Africa and as a consequence are therefore highly unlikely to seek medical attention. What is reported in these five studies illustrates that this indeed could only be the ‘tip of the iceberg’.

A holistic and integrated approach is required for women who have experienced sexual violence in South Africa. This needs to start with the police and linking with healthcare professionals within clinical and therapeutic settings and indeed was highlighted in the Lamola report (2017). This is not only a pressing need, not least highlighted with the widespread pandemic in South Africa but also necessary for the mental health and well-being of sexual violence survivors.

A comprehensive report conducted by Rohrs (2011) working with non-governmental organisations (NGOs) found a clear distinction between referral support information that can be provided by police officers to survivors and the role of healthcare facilities and specialist TCC settings. The administering of PEP for the prevention of HIV infection is part of the National Instructions (Section 9, 12) and is a public right for those who have been sexually assaulted (Lamola 2017; Nare 2013). However, it is recognised that there is a currently low uptake and adherence (Lamola 2017; Nare 2013). It is therefore imperative that those engaging with survivors, including healthcare professionals, provide the appropriate advice and guidance about PEP adherence, including the administering of anti-nausea medication. A lack of information from healthcare professionals at the time of administering necessary drugs may be a reason for the low adherence rates in South Africa and the continued high prevalence of HIV and STIs (Rohrs 2011; Schneider et al. 2016).

It is clear that there is a distinct legal process in place for survivors to report incidents and to seek medical attention at either specialist TCC or at acute settings. However, there is still considerable stigma attached to rape and sexual assault, which is underreported and has the added complication of a possible positive HIV outcome. Evidence is clear that many women, who are HIV-positive, are also rape survivors (Meel 2003). This presents a challenge for public health authorities in South Africa for providing support that meets the needs of survivors of both sexual violence and HIV while also ensuring that follow on support is integrated and meaningful to the growing number of survivors who are now encouraged to report assault. South Africa finds itself with a triplicate of dilemmas. It is a country dominated by a history of HIV, a neglected healthcare system with a mixture of public and private provision, and a systemic acceptance of daily accounts of rapes.

## Strengths and limitations

This review is the first to synthesise any empirical research that explores women’s experiences of healthcare services following sexual violence in South Africa. Shortcoming of existing services have been highlighted. Given the prevalence of sexual violence in the country, this work is imperative for emphasising the need to improve services for the large number of women who experience sexual abuse and violence in South Africa. This includes reviewing the impact of the pandemic on service provision for survivors (Kaswa 2021). The number of included studies (*n* = 5) is limited because of a lack of published research in this area, and those that have been included have shown a robust research procedure and given a voice to their female participants which may not have otherwise been heard.

This review has not included studies that have focused predominately on HIV adherence by survivors of sexual violence or indeed ongoing interventions in relation to HIV offered by TCC, healthcare facilities or NGOs. Likewise, studies focusing on the legal system or police involvement in sexual violence disclosures have not been included. This review aimed to identify evidence whereby survivors of sexual violence have shared their experiences about contact with healthcare facilities as a means to describe women’s experiences in South Africa, which is often neglected and currently lacking in the literature. Future work could benefit from a broader inclusion of women’s experiences with police, the legal systems and follow-up services and how these may impact pursuit of medical and psychological support by women.

## Future directions and implications for clinical practice

In addition to the physical care that the victims are given, there is a need to provide the concomitant caring and emotional support needed during this period of confusion and to address the assault to human dignity on the patient who is a victim of sexual violence. Healthcare professionals have an expressed duty to treat the patient with respect, to be non-judgemental and to foster a caring nurse–patient relationship (Randa & Mokoena 2019). Healthcare practitioners should also offer the victims of sexual violence information on how and where they can access legal aid and counselling services. This calls for the need for an integrated multisectoral approach and holistic patient-centred care, emphasising the importance of attending follow-up for the survivors of sexual violence.

## Conclusion

South Africa has conducted and published a few studies to date that have explored the survivor experiences of sexual violence. As such, this review encapsulates the multifaceted phenomenon of sexual violence within the particular context of South Africa from the perspective of women themselves. Geographically, it is also timely to remember that this is by no means unique, as sexual violence remains a significant global problem. With the inception of specialist care provision, TCCs for women who have experienced sexual violence in South Africa, these services have developed over the last four decades. However, as the present review highlights, alongside immediate care facilities, there needs to be a greater emphasis on sustained care pathways, combining mental health and psychological support for women post trauma. This requires substantial investment and resources, both financial support and wider resources including physical infrastructure and training. However, alongside any frontline service or care delivery development, the wider context of policy reform and systemic societal change with regard to prevailing assumptions and attitudes towards GBV need to be considered and are pivotal to this evolving health agenda. Further empirical work is required to fully understand and meet the needs of this often-hidden population. Given the stigma and societal complexities surrounding sexual violence, this needs to be understood through the lens of those who have experienced sexual harm. Post the COVID-19 pandemic, there is a duty to address this so-called ‘shadow pandemic’ not just in South Africa but globally (UN 2020).

### Critical findings

The voices of the women are often overlooked and neglected. This review indicates that more work is required if appropriate and timely support is to be provided. Sexual violence is a global issue, but healthcare in South Africa is fundamentally seen as a place of medical assistance rather than emotional or psychological support. There is a paucity of evidence, from disclosure through to the conviction of perpetrators, as a whole scale approach to tackling such issues for women. South Africa finds itself with a triplicate of dilemmas. This is a country dominated by a history of human immunodeficiency virus (HIV), a neglected healthcare system with a mixture of public and private provision, and a systemic acceptance of daily accounts of rapes.

### Implications for practice, policy and research

This is the only published review that synthesises the views of women who have experienced sexual violence in South Africa and sought healthcare assistance. There is evidence of robust legislation and protocols in place for survivors of sexual violence in South Africa; however, there are notable gaps between police and healthcare, which can result in women being left unsupported and therefore unlikely to report, especially if not accessing a Thuthuzela Care Centre (TCC). There is a need to review the role of the TCC and adopt a clear and defined route for any survivor of sexual violence to avoid the acceptance of the deficit model of care. Research that considers all aspects of the survivor’s journey and explores an inclusive understanding, inside and outside of TCC provision, needs to be undertaken. A holistic and integrated approach is integral if South Africa is to move beyond reactive to a proactive approach to tackle this continuing concern.
